# Traffic-Related Air Pollution and QT Interval: Modification by Diabetes, Obesity, and Oxidative Stress Gene Polymorphisms in the Normative Aging Study

**DOI:** 10.1289/ehp.0901396

**Published:** 2010-03-01

**Authors:** Emmanuel S. Baja, Joel D. Schwartz, Gregory A. Wellenius, Brent A. Coull, Antonella Zanobetti, Pantel S. Vokonas, Helen H. Suh

**Affiliations:** 1 Department of Environmental Health, Harvard School of Public Health, Boston, Massachusetts, USA; 2 Department of Community Health, Brown University, Providence, Rhode Island, USA; 3 Department of Biostatistics, Harvard School of Public Health, Boston, Massachusetts, USA; 4 Veterans Affairs Normative Aging Study, Veterans Affairs Boston Healthcare System, Boston, Massachusetts, USA; 5 Department of Internal Medicine, Boston University, Boston, Massachusetts, USA

**Keywords:** air pollution, diabetes, distributed lags, genes, obesity, oxidative stress, QT interval, smoking, traffic

## Abstract

**Background:**

Acute exposure to ambient air pollution has been associated with acute changes in cardiac outcomes, often within hours of exposure.

**Objectives:**

We examined the effects of air pollutants on heart-rate–corrected QT interval (QTc), an electrocardiographic marker of ventricular repolarization, and whether these associations were modified by participant characteristics and genetic polymorphisms related to oxidative stress.

**Methods:**

We studied repeated measurements of QTc on 580 men from the Veterans Affairs Normative Aging Study (NAS) using mixed-effects models with random intercepts. We fitted a quadratic constrained distributed lag model to estimate the cumulative effect on QTc of ambient air pollutants including fine particulate matter ≤ 2.5 μm in aerodynamic diameter (PM_2.5_), ozone (O_3_), black carbon (BC), nitrogen dioxide (NO_2_), carbon monoxide (CO), and sulfur dioxide (SO_2_) concentrations during the 10 hr before the visit. We genotyped polymorphisms related to oxidative stress and analyzed pollution–susceptibility score interactions using the genetic susceptibility score (GSS) method.

**Results:**

Ambient traffic pollutant concentrations were related to longer QTc. An interquartile range (IQR) change in BC cumulative during the 10 hr before the visit was associated with increased QTc [1.89 msec change; 95% confidence interval (CI), −0.16 to 3.93]. We found a similar association with QTc for an IQR change in 1-hr BC that occurred 4 hr before the visit (2.54 msec change; 95% CI, 0.28–4.80). We found increased QTc for IQR changes in NO_2_ and CO, but the change was statistically insignificant. In contrast, we found no association between QTc and PM_2.5_, SO_2_, and O_3_. The association between QTc and BC was stronger among participants who were obese, who had diabetes, who were nonsmokers, or who had higher GSSs.

**Conclusions:**

Traffic-related pollutants may increase QTc among persons with diabetes, persons who are obese, and nonsmoking elderly individuals; the number of genetic variants related to oxidative stress increases this effect.

Evidence from epidemiologic studies shows a consistent association between increased ambient air pollution and increased daily hospital admission (Lee et al. 2007; Santos et al. 2008; Schwartz 1999; Zanobetti and Schwartz 2006) and premature death (Pope et al. 2004; Schwartz 1994). Studies have further shown that the pollution-mediated impacts were largest for cardiovascular-related illness and deaths (von Klot et al. 2009; Zanobetti and Schwartz 2007). Several biological mechanisms by which air pollution can elicit cardiovascular morbidity and mortality have been identified, including oxidative stress (Gurgueira et al. 2002), autonomic dysfunction (Gold et al. 2000), and systemic inflammation (Peters et al. 2001b; Ruckerl et al. 2006), leading to endothelial dysfunction (O’Neill et al. 2005), atheromatous plaque (Sun et al. 2005; Suwa et al. 2002), and thrombosis (Baccarelli et al. 2008). However, the specific underlying biological pathways are not fully understood, and the identification of these pathways warrants further study.

Of particular interest are recent studies that suggest that myocardial infarctions risk (Peters et al. 2001a, 2004), inflammation (Ruckerl et al. 2006), and cardiac repolarization changes (Henneberger et al. 2005; Yue et al. 2007) may be associated with exposures to air pollution on time scales of less than a day. Because standards for particles and nitrogen dioxide (NO_2_) currently involve longer-term averages, these studies suggest that the relation of hourly peaks in air pollution with indicators of cardiovascular health should be examined.

Of the cardiovascular health indicators, irregularities in myocardial repolarization may be especially important because they can lead to the development of cardiac arrhythmias. Two panel studies of patients with cardiovascular disease, which were conducted in East Germany, showed evidence of an immediate effect of air pollution on ventricular repolarization duration, morphology, and variability (Henneberger et al. 2005; Yue et al. 2007). Ljungman et al. (2008) found that patients with implantable cardioverter defibrillators also showed evidence of rapid effects of air pollution on the risk of life-threatening ventricular arrhythmias. These findings suggest a possible biological pathway linking an acute effect of air pollution on increased risk of ventricular repolarization, cardiovascular arrhythmias, and cardiac death. However, the effect of air pollution on repolarization parameters in other populations at risk should be addressed and confirmed.

We hypothesized that short-term exposures to traffic-related air pollutants may be associated with increases in ventricular repolarization, as measured by changes in the heart-rate–corrected QT interval (QTc) on the electrocardiogram (ECG), and that participant characteristics and genes related to oxidative stress may modify this association. Thus, in this study, we investigated the association between ambient air pollution [black carbon (BC), carbon monoxide (CO), NO_2_, ozone (O_3_), PM ≤ 2.5 μm in aerodynamic diameter (PM_2.5_), and sulfur dioxide (SO_2_)] and a measure of ventricular repolarization, the QTc, among men residing in communities in the Boston, Massachusetts (MA), area to examine whether this association is modified by participant characteristics and by genetic susceptibility to oxidative stress. We evaluated these aims using data from a prospective longitudinal study of 580 elderly male participants of the Veterans Affairs (VA)Normative Aging Study (NAS).

## Materials and Methods

### Study population

The NAS is an ongoing longitudinal study of aging, which was established in 1963 by the Veterans Administration (Bell et al. 1972). Briefly, 2,280 community-dwelling, healthy men living in the Greater Boston area were enrolled between 1963 and 1968. Every 3–5 years, participants visited the VA Medical Center NAS clinic after an overnight fast and abstention from smoking for an extensive physical examination, laboratory tests, blood collection, and a self-administered questionnaire on alcohol consumption, food intake, medical history, medication use, smoking history, and other factors that could affect health. From November 2000 to December 2008, ECG measurements were also obtained during each participant’s regularly scheduled visit. To date, ECG measurements have been collected for 712 participants. Of these, we excluded 132 participants because the recording time of the ECG measurements was < 3.5 min, the T-wave amplitude was insufficient, or the heart beats were nonnormal or nonsupraventricular. The remaining 580 men had one (*n* = 318), two (*n* = 178), or three (*n* = 84) ECG measurements, for a total of 926 valid readings. All participants gave written consent, and we received institutional review board approval for this study.

### ECG measurement and analysis

The ECG was recorded for 5–10 min between 0530 and 1400 hours with a two-channel (five lead) ECG monitor (Trillium 3000; Forest Medical, Inc., East Syracuse, NY) using a sampling rate of 256 Hz per channel. A detailed description of the protocol is provided elsewhere (Park et al. 2005; Pope et al. 2001). Briefly, beats were automatically labeled, and the QT interval was measured from the QRS onset to the end of the T-wave only on normal or supraventricular beats. The QT interval was not calculated if the T-wave did not have sufficient amplitude. The QTc values were calculated using the Bazett’s formula (Bednar et al. 2001). We calculated the mean QTc for the length of the recording at the time of each participant’s visit.

### Air pollution and meteorology

Ambient BC and PM_2.5_ concentrations were continuously measured at the stationary ambient monitoring site at the Harvard University Countway Library, which is located < 1 km from the clinical laboratory where subjects were examined. Hourly ambient CO, O_3_, NO_2_, and SO_2_ concentrations were obtained from several local monitoring sites of the Massachusetts Department of Environmental Protection. We used the following locations to calculate the mean concentrations of the gases: Roxbury, Bremen, Kenmore Square, the North End of Boston, Lynn, and Waltham, MA. The distance of the sites from the clinical laboratory varied from < 2 km to < 20 km. Temperature data were obtained from the weather station at Boston Logan airport.

### Genotyping and genetic susceptibility score

Participants were genotyped for two gene deletions [glutathione *S*-transferase mu-1 (*GSTM1*) and glutathione *S*-transferase theta-1 (*GSTT1*)], eight single-nucleotide polymorphisms (SNPs) [glutathione *S*-transferase pi-1 (*GSTP1*) Ile105Val and Ala114Val, hemochromatosis (*HFE*) C282Y and H63D, NAD(P)H dehydrogenase, quinone-1 (*NQO1*) C609T, and catalase (*CAT*) RS2300181, RS2284367, and RS769217], and a microsatellite GTn-repeat polymorphism [heme oxygenase (decycling)-1 (*HMOX1*)]. The dominant genetic model was used to classify the SNPs of the participants. The SNPs were categorized as either a wild type (WT) or any variant [for a detailed description, see Supplemental Material (doi:10.1289/ehp.0901396)].

To reduce multiple comparisons by investigations of multiple gene deletions and polymorphisms, we created a variable for the genetic susceptibility score (GSS) that was related to the genetic susceptibility of a participant to oxidative stress. The GSS assumed that all the deletions and polymorphisms have an equal weight contribution to the genetic susceptibility of a participant to oxidative stress. A genotype was defined as unfavorable based on findings from the scientific literature of the polymorphisms or of gene–air pollution interactions. Catalase SNPs were not included in the scoring method because of the lack of prior studies reporting their effects. The role of the proteins encoded by these genes in the oxidative stress pathway is summarized in Supplemental Material, Table 1 (doi:10.1289/ehp.0901396). For every gene null deletion, SNP, or microsatellite GTn-repeat polymorphism, a score of 1 was given if the participant had the unfavorable genotype and 0 if the participant did not have this genotype. The scores were added for every participant. Based on the observed distribution of the score, the GSS of every participant was categorized into low [GSS < 4 (< distributional median)] and high [GSS ≥ 4 (above median)] susceptibility to oxidative stress.

### Statistical analysis

We calcuated descriptive statistics for QTc, air pollutants, and covariates, as well as correlation coefficients to evaluate the relationship among QTc, covariates, and air pollutants.

Associations between each air pollutant and the change in mean QTc were estimated using linear mixed-effects models with random subject-specific intercepts, which is a standard approach for analyzing longitudinal data with repeated measures on the same subject (Fitzmaurice et al. 2004). Mixed models with a random subject-specific intercept were fit to capture residual correlation among measurements within the same participant and to account for the heterogeneity in the subject’s overall QTc.

In all models we controlled for age, body mass index (BMI), sitting mean arterial blood pressure (MAP), cholesterol level, alcohol intake, smoking status, diabetic status, and cardiac medication use (alpha blockers, angiotensin-converting enzyme inhibitors, angiotensin receptor antagonist/blockers, beta-blockers, and calcium channel blockers). These covariates were chosen *a priori* as potentially important predictors of QTc. To account for seasonal variation and other long-term time trends in QTc, we used a natural spline of calendar date with 4 degrees of freedom per year. We also controlled 1-hour mean temperature using a linear term and modeled day-of-the-week effects with indicator variables.

Separate single-pollutant models were evaluated. To examine the appropriate exposure window for each pollutant and to minimize multiple comparisons, our primary analysis fit a quadratic constrained distributed lag (QCDL) model to estimate the cumulative effect of each pollutant during a 10-hr time window before the visit (Schwartz 2000), which, using the Akaike information criterion, gave the best model fit for the cumulative effect of the pollutant. We verified the results of the QCDL model by fitting separate hourly exposure lag models for each lag. The fixed covariates were included in all analyses.

To evaluate obesity as an effect modifier, subjects were classified into two groups according to BMI (obese, ≥ 30 kg/m^2^; nonobese, < 30 kg/m^2^). We also assessed effect modification by a history of diabetes [diabetic vs. nondiabetic: doctor’s diagnosis of disease or fasting blood glucose (FBG) > 126 mg/dL vs. no disease diagnosis or FBG ≤ 126 mg/dL)], smoking (ever smoker vs. never smoker), and genetic susceptibility to oxidative stress (high vs. low GSS, ≥ 4 vs. < 4). In addition, we assessed effect modification by each gene deletion and polymorphism to check if these deletions and polymorphisms individually modify the associations of air pollutants with QTc. We included in the models interaction terms between the dichotomized modifier variable and each air pollutant. The likelihood ratio test (LRT) was used to test the significance of the interaction terms.

Effect size estimates were reported as change in mean QTc per interquartile range (IQR) change of a pollutant. In addition, effect estimates were also scaled to percentage of a standard deviation (SD) change in QTc per IQR change of a pollutant and are detailed in the Supplemental Material (doi:10.1289/ehp.0901396). We considered *p*-values of ≤ 0.05 to be statistically significant. R (version 2.8.1; R Foundation for Statistical Computing 2008), SAS (version 9.1) and and JMP (version 8; SAS Institute Inc., Cary, NC) were used in the analysis [see Supplemental Material (doi:10.1289/ehp.0901396)].

## Results

Eligible study participants included 580 NAS participants, who had a total of 926 valid ECG recordings available for analysis. The participants were older men with a mean age (± SD) of 74.8 years ± 6.8 years who were generally overweight with a mean BMI (± SD) of 27.9 kg/m^2^ ± 4.1 kg/m^2^. Table 1 shows other participant characteristics.

Table 2 shows the descriptive statistics of pollutant concentrations and temperature. All pollutant concentrations during the actual hour of ECG monitoring (0-hr lag) were significantly correlated with each other, with BC and PM_2.5_ having the highest correlation (Spearman correlation coefficient, ρ = 0.69). Among the gaseous pollutants, NO_2_ and CO were most strongly correlated (ρ = 0.64), whereas the correlation between particulate and gaseous pollutants was highest for BC and NO_2_ (ρ = 0.60). In contrast, O_3_ was negatively correlated with every other pollutant (ρ < −0.12).

**Figure 1:**
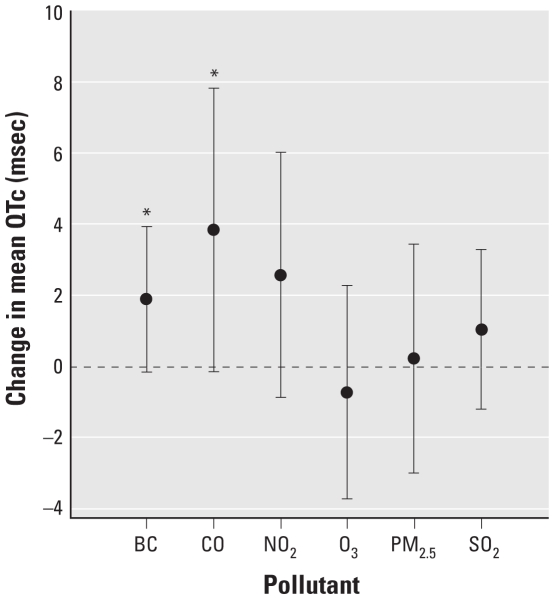
Effect estimates of change in mean QTc per IQR change in cumulative exposure to pollutant during the 10 hr before ECG measurement: single-pollutant model with random intercept using QCDL. All models were adjusted for age; BMI; MAP; cholesterol; diabetic status (doctor’s diagnosis of disease or FBG > 126 mg/dL vs. no diagnosis or FBG ≤ 126 mg/dL); alcohol intake (≥ 2 drinks/day, < 2 drinks/day); cigarette smoker (never, ever); use of alpha blockers, angiotensin-converting enzyme inhibitors, angiotensin receptor antagonist/blockers, beta blockers, or calcium channel blockers; day of the week (Monday through Sunday); temperature; and a natural spline for long-term time trend (date). Error bars indicate 95% CI. *p < 0.10.

Figure 1 shows the estimated cumulative effect of each pollutant for exposures for the 10 hr before the QTc measurement using the QCDL model. BC, CO, and NO_2_ were positively associated with QTc, but the results were not statistically significant [BC: 1.89 msec change in mean QTc; 95% confidence interval (CI), −0.16 to 3.93; *p*-value = 0.07; CO: 3.83 msec change in mean QTc; 95% CI, −0.17 to 7.82; *p*-value = 0.06; NO_2_: 2.56 msec change in mean QTc; 95% CI, −0.88 to 6.00; *p*-value = 0.14]. In contrast, PM_2.5_, O_3_, and SO_2_ were not clearly associated with QTc [see Supplemental Material, Table 2 (doi:10.1289/ehp.0901396)].

**Figure 2:**
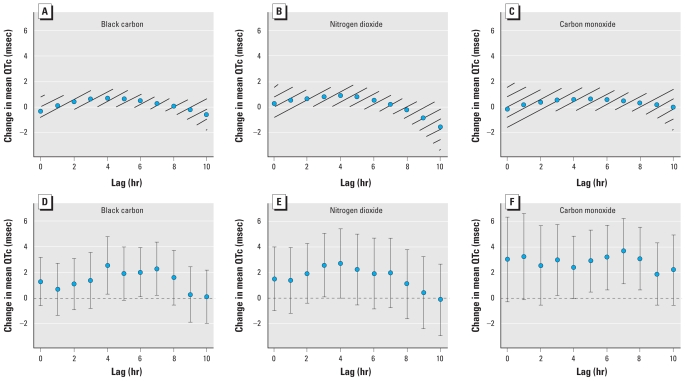
Effect estimates of change in mean QTc per IQR change of traffic-related pollutant. Error bars and hatch-marked regions indicate 95% CI. (*A*–*C*) Single-pollutant model of cumulative exposure for a 10-hr time window with random intercept using QCDL with hourly lags: BC (*A*), NO_2_ (*B*), and CO (*C*). (*D*–*F*) Single-pollutant model of hourly exposure with random intercept using hourly lags: BC (*D*), NO_2_ (*E*), and CO (*F*).

In Figure 2, we compare the results for cumulative exposure for a 10-hr time window (based on QCDL models) and hourly exposure lag models. The two approaches showed an increase in QTc during a similar time course for BC, NO_2_, and CO. For BC, the strongest associations were observed with pollutant measures 4 hr before the ECG measurement. IQR changes in BC, NO_2_, and CO in the 4th hr were associated with a 2.54 msec (95% CI, 0.28–4.80), 2.72 msec (95% CI, −0.01 to 5.44), and 2.36 msec (95% CI, −0.07 to 4.80) change in mean QTc, respectively. Not all of the measures were statistically significant, but IQR changes in BC, NO_2_, and CO at other hourly lags were also related to longer QTc. This finding shows a consistent trend across hourly lags. In contrast, we found no significant association between QTc and ambient PM_2.5_, SO_2_, and O_3_ [see Supplemental Material, Table 2 (doi:10.1289/ehp.0901396)].

### Effect modification by diabetes, obesity and smoking

We assessed whether being diabetic, obese, or a smoker modified the effect of BC, NO_2_, and CO on QTc. 6% of the study population were obese, diabetic, and nonsmoker, 8% were obese and diabetic, 6% were nonsmokers and obese, and 5% were nonsmokers and diabetic.

**Figure 3:**
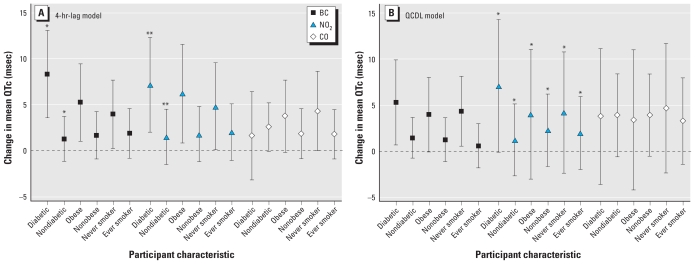
Adjusted change in mean QTc per IQR change of traffic-related pollutant: hourly exposure with a 4-hr lag (*A*) or cumulative exposure during 10 hr before ECG measurement (*B*), by participant characteristics [diabetic status (doctor’s diagnosis of diabetes or FBG > 126 mg/dL, vs. no diagnosis or FBG ≤ 126 mg/dL), smoking (never, ever), and obesity (BMI ≥ 30, < 30)]. Error bars indicate 95% CI. *p-Value interaction < 0.01; **p-Value interaction < 0.05.

Figure 3 shows the results of effect modification by diabetes, obesity, and smoking for both the QCDL model (for cumulative exposure during the 10 hr before QTc measurement) and the hourly exposure model (with a 4-hr lag) for BC, NO_2_, and CO. The estimated effect of BC on QTc is consistent for both models. Cumulative BC exposure was associated with a 5.28 msec (95% CI, 0.67–9.90) change in mean QTc among participants with diabetes, but we estimated a smaller association for nondiabetic participants (1.43 msec change in mean QTc; 95% CI, −0.79 to 3.65; *p*-value for interaction = 0.26 using the LRT). Additionally, the association with cumulative BC was also stronger for obese versus nonobese participants and never-smokers versus ever-smokers, although differences between estimates were not statistically significant (LRT, *p*-values for interaction > 0.53; for details, see Table 3).

### Effect modification by GSS

We also assessed whether having a high GSS (unfavorable genotype) modified associations of traffic-related pollutants with QTc; 94%, 90% and 85% of the participants in the “high polymorphism” group had the unfavorable polymorphism of *HFE* C282Y, *GSTP1* A114V, and *HFE* H63D, respectively.

**Figure 4:**
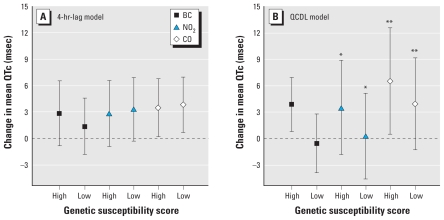
Adjusted change in mean QTc per IQR change of traffic-related pollutant: hourly exposure with a 4-hr lag (*A*) or cumulative exposure during 10 hr before ECG measurement (*B*), by GSS (low GSS, high GSS). Error bars indicate 95% CI. *p-Value interaction < 0.01; **p-value interaction < 0.10.

Figure 4 shows the results of the analysis of effect modification by GSS for BC, NO_2_, and CO. Cumulative BC exposure (over the previous 10 hr) was associated with a 3.85 msec (95% CI, 0.78–6.93) increase in mean QTc in participants with a high GSS, but no association was evident among participants with a low GSS (−0.56 msec; 95% CI, −3.90 to 2.78; LRT, *p*-value for interaction = 0.57; for details, see Table 4).

## Discussion

This study provides evidence that the traffic-related pollution markers BC, CO, and NO_2_ are associated with prolongation of QTc, a marker of ventricular repolarization and a risk factor for ventricular arrhythmias (e.g., torsades de pointes) and sudden cardiac death. In contrast, we found no significant associations with PM_2.5_, O_3_, or SO_2_, generally indicators of nontraffic pollution. Our results further suggest that diabetic, nonsmoker, and obese participants and individuals with a high number of unfavorable genotypes related to oxidative stress may be particularly at risk from traffic-related exposures.

Our findings are supported by those from previous studies that also show evidence of greater cardiovascular effects for traffic-related pollutants than for regional nontraffic-related pollutants (Laden et al. 2000, 2006; Schwartz et al. 2005a). In the Six City Study, for example, the estimated effect of particles from mobile sources on mortality was greater than that from coal power plants (Laden et al. 2000, 2006). Consistent with these findings, ambient BC concentrations have been associated in several Boston-area studies with mortality (Maynard et al. 2007) as well as a variety of intermediate cardiovascular indicators, including heart rate variability (Schwartz et al. 2005a), ST-segment depression (Chuang et al. 2008; Gold et al. 2005), and homocysteine (Park et al. 2008).

Few studies have been conducted that have examined pollution-mediated effects on QTc. In a prospective panel study in East Germany, Henneberger et al. (2005) examined pollution-induced repolarization changes in 56 males (mean age, 66 years) with ischemic heart disease. They found no significant association at alpha = 0.05 between QTc and elemental carbon, but found significant associations with NO_2_ and CO during 6–11 hr and with SO_2_ during the whole 0–23 hr before ECG recording. Insignificant findings for EC in the East Germany study may be due to its smaller sample size or its younger study population compared with our study, in which we estimated significant main effects of BC 4 hr before ECG recording (Figure 2) in our population of 580 men with a mean age of 74.8 years.

### Plausible pathophysiologic mechanisms

Our observed associations can be explained by plausible pathophysiological mechanisms. Toxicity of traffic particles may have a direct effect on the blood, cardiovascular system, and lung receptors (Brook et al. 2004; Simkhovich et al. 2008). Deposition in the airways and lung alveoli may trigger proinflammatory signaling via a reactive oxygen species (ROS)-dependent mechanism (Donaldson and Stone 2003; Ghio and Devlin 2001; Ghio et al. 2000; Li et al. 1999). Moreover, traffic particles may also cross the pulmonary epithelium and may be able to reach the heart via the vasculature (Nemmar et al. 2001, 2002; Oberdörster et al. 2002), where they may induce oxidative stress and proinflammatory changes in the vasculature and myocardial substrate (Simkhovich et al. 2008). The generated proinflammatory cytokines and ROS may subsequently affect a variety of health measures, including autonomic cardiac control (Simkhovich et al. 2008). Changes in autonomic control may in turn cause a prolongation in ventricular repolarization and QTc interval through the altering of the function of the sodium and calcium channels (Goldenberg et al. 2008; Moss and Kass 2005; Utell et al. 2002; Zareba et al. 2001).

### Effect modification by participant characteristics

Our observation of effect modification by diabetic status in the associations between the cumulative effect and 4 hr before ECG measurement lag effect of BC and NO_2_ exposure and prolonged QTc for the QCDL and hourly lag models may be due to a higher likelihood of persons with diabetes to have prolonged QTc than those without diabetes. In addition, the greater baseline oxidative stress among persons with diabetes has been shown to mediate the autonomic effects of particles (Chahine et al. 2007; Schwartz et al. 2005b; Rhoden et al. 2005). Both the QCDL and hourly lag models showed a consistent pattern in the acute estimated effects of BC and NO_2_ on QTc. These results are supported by those from a population based cohort study that showed a high prevalence of prolonged QTc among participants with type 2 diabetes (Veglio et al. 2002).

We also observed evidence of effect modification by obesity on associations between cumulative (for the 10 hr before ECG measurement) and hourly (lagged 4 hr before) BC and NO_2_ exposures and prolonged QTc. Obesity and the cardiac autonomic nervous system are related, an increase in body weight is associated with a decline in parasympathetic tone and accompanied by a rise in mean heart rate (Poirier et al. 2006). El-Gamal et al. (1995) have found that obese individuals are more likely to have prolonged QTc than nonobese individuals and a positive association exists between BMI and QTc. This may explain why we found a stronger association between BC and NO_2_, and QTc among obese participants compared with nonobese participants. Because only 8% of the participants were both obese and diabetic, it is unlikely in this case that obesity is acting as a surrogate for diabetes.

In addition, we observed evidence of effect modification by smoking status on the association between QTc and BC and NO_2_ exposures (both as the cumulative and 4-hr lag effect), with stronger associations estimated for nonsmokers. We hypothesize that nonsmokers are more susceptible to effects of traffic-related pollution, because particles and gases may penetrate the clear lungs of nonsmokers more easily than the lungs of smokers who have extensive lung damage. However, results from previous studies provide conflicting information on the effect of smoking on prolonged QT interval (Andrassy et al. 2003), because Ileri et al. (2001) reported that QTc was significantly prolonged in smokers than in nonsmokers. Clearly, more studies are needed to verify our observed effect modification by smoking. Nevertheless, our results indicate that persons with diabetes, individuals who are obese, and persons who do not smoke may be more responsive to traffic-related pollutants, such as BC and NO_2_, compared with those who are not diabetic, obese, and smokers.

### Effect modification by genetic susceptibility to oxidative stress

Our QCDL model estimates showed significant QTc prolongation in association with increasing BC and CO in 50% of the study population with higher genetic susceptibility to oxidative stress (as measured by GSS). In contrast, no associations with traffic pollutants were seen in half of the study population with lower GSS. Our findings are supported by several gene–environment interaction studies of our group that showed effect modification by genes related to oxidative stress (Chahine et al. 2007; Park et al. 2006; Schwartz et al. 2005b). The observed interactions between traffic pollutants and genetic susceptibility to oxidative stress score imply that components of traffic pollutants may play a significant role in influencing oxidative stress via changes in autonomic signaling, and changes in the levels of oxidants and antioxidants (Rhoden et al. 2005; Simkhovich et al 2008).

We hypothesize that participants with high polymorphisms related to oxidative stress are more susceptible to an increase in oxidant generation and/or a decrease in antioxidant protection and this susceptibility could lead to QTc prolongation. Oxidative stress could increase ROS in the endothelial cells and/or heart muscles that could induce endothelial dysfunction and myocardial inflammation. Endothelial dysfunction and inflammation could alter the function of ion channels leading to an intracellular excess and accumulation of positively charged ions inside the cardiac myocytes, which extends ventricular repolarization and results in QT interval prolongation (Al-Khatib et al. 2003; Viskin 1999). Oxidative stress also inhibits nitric oxide production (Bai et al. 2001). Nitric oxide inactivation is associated with a reduction in parasympathetic tone and with an elevation in sympathetic tone (Chowdhary et al. 2002), which could trigger QTc prolongation and lead to arrhythmia, and sudden cardiac death (Tapanainen et al. 2002; Tsuji et al. 1996).

Our study used the GSS method to assess if having a higher number of genetic variants related to oxidative stress modified the associations between traffic-related pollutants and QTc. This approach is a statistically efficient method to evaluate gene–environment interactions especially in inferring biological pathways that would link exposure to outcome of interest. The use of the score method approach, together with the distributed lag models, reduces the problem of multiple comparisons. Results from the GSS method were consistent with those from the analysis of individual genetic polymorphisms [see Supplemental Material (doi:10.1289/ehp.0901396)]. Nevertheless, more studies are needed to verify this approach.

### Strengths and limitations

One limitation of our study is the use of a single ambient monitoring site as a surrogate for recent ambient pollution exposure, which will not capture spatial variation in air pollutant concentrations. This spatial variation may result in exposure misclassification because participants lived 22 km on a median straight line distance from our ambient monitoring site. This exposure misclassification would probably be nondifferential and bias the results toward the null (Sarnat et al. 2005).

Unlike previous studies that read the ECG recordings manually, our study used a predefined computer algorithm that automatically detected beat labels and QTc intervals to analyze ECG recordings. This made the outcome assessment of QTc less susceptible to differential measurement errors and intertechnician variability. In addition, the use of a random subject intercept means that contrasts were predominantly within and not between subjects. Hence, confounding factor bias should be less than that incurred in a purely cross-sectional design, although bias due to residual or unmeasured confounding cannot be ruled out.

Another limitation of the study is the assumption that the null deletions and polymorphisms have equal “weight” contribution to the genetic susceptibility of a participant to oxidative stress. This assumption does not take into account the possible interaction between genes that may or may not affect the genetic susceptibility of a participant to oxidative stress.

Because the study population consists of older men who are predominantly white, the results may not be generalizable to women, younger individuals, or to other racial and ethnic groups. The effect of traffic pollutants on QTc on these other populations should be addressed in future studies.

## Conclusions

In summary, this study documents the association between elevated short-term exposure to traffic-related pollution and prolonged QTc, a marker of ventricular arrhythmias commonly associated with heart attack, among older men. The study also showed that traffic-related pollutants is related to prolonged QTc, given the generally significant association findings for BC, NO_2_, and CO (Alm et al. 1999; Chan et al. 1991). Furthermore, older men with few genetic variants related to oxidative stress appear to be better protected from QTc prolongation with traffic exposure than older men with high genetic variants related to oxidative stress. Moreover, the study provides further evidence that traffic pollutants via the oxidative stress pathway may play a crucial role in cardiopulmonary toxicity.

## Figures and Tables

**Table 1:** Characteristics of the study population (NAS participants, *n* = 580).

Characteristic	Value
Age [years (mean ± SD)]	74.8 ± 6.8
BMI [kg/m^2^ (mean ± SD)]	27.9 ± 4.1
≥ 30 kg/m^2^ (%)	27.6
MAP [mmHg (mean ± SD)]	90.0 ± 11.1
Cholesterol [mg/dL (mean ± SD)]	246.2 ± 131.2
Alcohol intake ≥ 2 drinks/day (%)	19.5
Ever diabetic (%)^a^	22.9
Cigarette smoking (%)^b^	
Former	69.0
Never	28.4
Current	5.0
Medication use (%)	
Beta blockers	42.9
Angiotensin-converting enzyme inhibitors	30.9
Alpha blockers	17.6
Calcium blockers	17.6
Angiotensin receptor antagonist/blockers	9.3
Genotypes (%)	
*HFE* C282Y WT^c^	82.6
*GSTP1* Ala114Val WT^c^	80.0
*HFE* H63D WT^c^	72.8
*CAT* rs769217 WT^c^	51.0
*CAT* rs2284367 WT^c^	50.7
*GSTM1* deletion^d^	49.0
*HMOX1* long/long alleles (≥ 25 GT repeats)	46.2
*GSTP1* Ile105Val any variant^e^	45.7
*CAT* rs2300181 any variant^e^	41.6
*NQO1* C609T any variant^e^	29.5
*GSTT1* deletion^d^	16.7
QTc interval [msec (mean ± SD)]^f^	393.3 ± 29.7

a Report of doctor’s diagnosis of disease or FBG > 126 mg/dL.

b Total does not equal 100% because of changes in smoking habit.

c Homozygous WT.

d Homozygous null.

e Heterozygous + homozygous variant.

f Bazett corrected, mean QTc interval.

**Table 2:** Concentration of ambient air pollutants and temperature during or 10 hr before ECG monitoring (November 2000–December 2008).

Variable	HR Lag	Mean ± SD	Median	IQR
BC (μg/m^3^)	0	1.08 ± 0.84	0.85	0.85
	10	0.64 ± 0.51	0.50	0.55
CO (ppm)	0	0.436 ± 0.299	0.381	0.302
	10	0.332 ± 0.280	0.266	0.271
NO_2_ (ppm)	0	0.021 ± 0.008	0.020	0.010
	10	0.019 ± 0.009	0.018	0.013
PM_2.5_ (μg/m^3^)	0	11.42 ± 8.55	9.03	8.56
	10	10.72 ± 7.88	8.75	7.92
O_3_ (ppm)	0	0.023 ± 0.016	0.021	0.018
	10	0.021 ± 0.015	0.020	0.020
SO_2_ (ppm)	0	0.0052 ± 0.0045	0.0038	0.0039
	10	0.0042 ± 0.0037	0.0030	0.0030
Temperature (°C)^a^		13.03 ± 9.65	13.33	14.96

a Current 1-hr mean temperature

**Table 3:** Adjusted effect estimates for change in mean QTc with an IQR increase in traffic pollutant exposures (cumulative during the 10 hr before ECG measurement) by patient characteristics (diabetic, obesity, and smoking status).

		QCDL model		
Status/pollutant	Modifier	Change in mean QTc [msec (95% CI)]	*p*-Value	*p*-Value interaction
Diabetic status				
BC	DM	5.28 (0.67 to 9.90)	0.03	0.26
	Non-DM	1.43 (−0.79 to 3.65)	0.21	
NO_2_	DM	7.10 (−0.12 to 14.32)	0.054	< 0.01
	Non-DM	1.22 (−2.67 to 5.12)	0.54	
CO	DM	3.78 (−3.59 to 11.14)	0.31	0.15
	Non-DM	3.91 (−0.62 to 8.44)	0.09	
Obesity status				
BC	Obese	3.97 (−0.07 to 8.01)	0.054	0.54
	Nonobese	1.23 (−1.14 to 3.60)	0.31	
NO_2_	Obese	4.02 (−3.01 to 11.04)	0.26	< 0.01
	Nonobese	2.28 (−1.65 to 6.21)	0.25	
CO	Obese	3.38 (−4.27 to 11.02)	0.38	0.29
	Nonobese	3.90 (−0.57 to 8.37)	0.09	
Smoking Status				
BC	Never	4.32 (0.54 to 8.09)	0.03	0.74
	Ever	0.57 (−1.83 to 2.98)	0.64	
NO_2_	Never	4.18 (−2.41 to 10.77)	0.21	< 0.01
	Ever	1.97 (−2.02 to 5.95)	0.33	
CO	Never	4.67 (−2.33 to 11.68)	0.19	0.14
	Ever	3.27 (−1.41 to 7.96)	0.17	

DM, diabetic (diagnosis of diabetes mellitus).

**Table 4:** Adjusted effect estimates for change in mean QTc with an IQR change in cumulative traffic pollutant exposure (during the 10 hr before ECG measurement) by genetic susceptibility to oxidative stress (high vs. low GSS).

		QCDL model		
Pollutant	Modifier	Change in Mean QTc [msec (95% CI)]	*p*-Value	*p*-Value interaction
BC	High GSS	3.85 (0.78 to 6.93)	0.01	0.57
	Low GSS	−0.56 (−3.90 to 2.78)	0.74	
NO_2_	High GSS	3.50 (−1.86 to 8.87)	0.20	< 0.01
	Low GSS	0.29 (−4.56 to 5.14)	0.90	
CO	High GSS	6.52 (0.49 to 12.55)	0.03	0.08
	Low GSS	3.94 (−1.28 to 9.16)	0.14	

GSS measures the genetic susceptibility of a participant to oxidative stress by adding all the unfavorable genotypes of the participant.
